# Improving Childhood Immunization Service Delivery in Cameroon: A Synthesis of Caregiver Experiences and Recommendations

**DOI:** 10.3390/vaccines12121430

**Published:** 2024-12-19

**Authors:** Yauba Saidu, Jessica Gu, Budzi Michael Ngenge, Sangwe Clovis Nchinjoh, Amani Adidja, Nnang Edwidge, Nkwain Muteh, Clarence Mbanga, Diaby Ousmane, Andreas Njoh, Junie Flegere, Demba Diack, Emanuele Montomoli, Sue Ann Costa Clemens

**Affiliations:** 1 Clinton Health Access Initiative Inc., Yaoundé P.O. Box 2664, Cameroon; 2 Institute for Global Health, University of Siena, 53100 Siena, Italy; 3 Global Vaccine Team, Clinton Health Access Initiative Inc., Boston, MA 02127, USA; 4 Faculty of Medicine and Biomedical Sciences, University of Yaoundé 1, Yaoundé 3077, Cameroon; 5 Gavi, the Vaccine Alliance, 1218 Geneva, Switzerland; 6 Department of Studies and Projects, Ministry of Public Health, Yaoundé 3038, Cameroon; 7 Expanded Program on Immunization, Cameroon Ministry of Public Health, P.O. Box 2084, Yaoundé 3038, Cameroon; 8 Department Molecular Medicine, University of Siena, 53100 Siena, Italy; 9 Department of Pediatrics, University of Oxford, Oxford OX1 2JD, UK

**Keywords:** caregiver, satisfaction, immunization experience, expanded program of immunization, Cameroon

## Abstract

Background/Objectives: A “people-centered” approach is one of the core principles of the Immunization Agenda (IA) 2030 and emphasizes the need for services to be organized around the needs and expectations of individuals and the community. A better understanding of the immunization experience from the client’s perspective is key to guiding the design of policies and interventions aimed at improving immunization delivery and coverage. This study provides a synthesis of the immunization experiences of children’s caregivers in Cameroon, highlighting potential barriers for timely and complete immunization. Methods: A descriptive cross-sectional study was conducted, targeting caregivers of children brought to selected health facilities for immunization in all ten regions of Cameroon. Using structured questionnaires, data were collected from caregivers and analyzed using STATA version 13. Results: In total, 1230 caregivers were interviewed in 265 health facilities. The median age of participants was 27 years and the median number of children per caregiver was two children. Most (87%) of the study participants reported to be satisfied with immunization service delivery. The median waiting time for vaccination was 1 h 48 min, with regional median waiting times ranging from 18 min in the South region to 4 h 6 min in the North region. About a quarter (24%) of surveyed participants reported to have presented to a health facility for immunization services and were turned away without achieving the purpose for which they came at least once. About half (48%) of the caregivers had never heard about planned vaccination activities in their communities. Conclusion: While most caregivers appeared to be satisfied with immunization service delivery in Cameroon, our study highlights some notable caregiver concerns (long waiting times, unproductive immunization visits and inadequate information about outreach activities) which, if addressed, may go a long way to enhance the immunization experience of caregivers in Cameroon, build trust in immunization services and thus improve vaccination uptake.

## 1. Introduction

Immunization is considered one of the “best buys” in global health, promoting health and wellbeing throughout the life course and contributing directly or indirectly to the achievement of all sustainable development goals [1,2,3]. Despite the multidimensional benefits of vaccination and the global progress made in improving vaccine coverage over the years, vaccine-preventable diseases (VPDs) remain a major cause of child morbidity and mortality, particularly in low- and middle-income countries (LMICs) [4,5]. Indeed, estimates suggest that approximately 30 million children under the age of five suffer from VPDs annually in Africa, with over half a million of these resulting in VPD-related deaths [6]. Most of these sufferings are, to a large extent, related to suboptimal immunization coverage. In 2019, nearly 20 million children were either un- or under-vaccinated for the third dose of diphtheria, tetanus and pertussis-containing (DTP-3) vaccine worldwide. Nearly 50% of these children were residing in the World Health Organization (WHO) African region [7], where a myriad of supply-side factors, including the quality of health service delivery, influence vaccine uptake.

There is growing evidence that poor quality of care is an important contributor to mortality in LMICs. According to WHO, approximately 5.7 to 8.4 million deaths annually can be attributed to poor quality of care in LMICs, accounting for up to 15% of mortality in these countries [8]. Negative experiences with poor-quality health care services can adversely affect a client’s relationship with the health system and lead to a reduction in their utilization of these services [9].

According to the WHO, quality immunization services are supposed to be safe, effective, timely, equitable, integrated, efficient and people-centered [9]. The latter, which is one of the core principles of the Immunization Agenda 2030 (IA2030), emphasizes the need for immunization services to be organized around the needs and expectations of individuals and the community, which in turn may lead to improved service experience and satisfaction [3]. A positive experience at points of service builds trust in immunization services and encourages utilization of the health system as a whole [9]. In contrast, patients and caregivers may refrain from using immunization services if they do not feel treated with value and respect, even when these services are accessible, undermining the numerous proven gains of immunization [10]. Several factors have been shown to contribute to immunization service experience, including health system (e.g., infrastructure, staffing, equipment and stock availability), provider (e.g., training and skills, attitude and interpersonal communication) and caregiver/client factors [11]. A better understanding of the immunization experience from the client’s or caregiver’s perspective is key to guiding the design of policies and interventions aimed at improving immunization delivery and coverage.

Few studies have explored client satisfaction with immunization services conducted in sub-Saharan Africa and many of them have shown great variations in client satisfaction across the region. For instance, a population-based study in Zambia reported a satisfaction rate of 20% [12]. Similarly, in Nigeria, Uwaibi et al. reported caregiver satisfaction rate of 19.4% with child immunization services [13]. The main reasons for dissatisfaction with immunization services in these studies included long waiting times, the poor attitude of vaccination staff and poor quality of communication between staff and caregivers/clients.

Although remarkable progress has been made in immunization uptake in Cameroon since the launch of the Expanded Program of Immunization (EPI) in 1976 [14], immunization coverage still falls below national targets. In Cameroon’s comprehensive multi-year plan (cMYP) for 2015 to 2019, the EPI envisioned raising the DTP-3 coverage from 89% in 2013 to 92% by 2019; however, coverage for this antigen plummeted by 22 points, falling from 89% in 2013 to 67% in 2019 [15,16]. A few studies have examined the performance of some components of Cameroon’s immunization system [17,18,19,20,21,22]; however, there is paucity of data on the immunization experiences of children’s caregivers in Cameroon. This study thus examined and provided a synthesis of the immunization experiences of children’s caregivers (parents, guardians, family members accompanying children to vaccination sessions) in Cameroon, highlighting potential barriers for timely and complete immunization.

## 2. Materials and Methods

### 2.1. Study Design and Setting

This was a descriptive study nested in a national baseline assessment carried out by the EPI and Clinton Health Access Initiative (CHAI). The study was designed to identify factors contributing to declining immunization coverage in Cameroon, a country in Central Africa, with a population of approximately 28 million inhabitants [23].

Politically, Cameroon is divided into 10 administrative regions: Adamawa (AD), Centre (CE), East (ES), Far-North (EN), Littoral (LT), North (NO), North-West (NW), West (OU), South (SU) and South-West (SW) regions [24]. Its health sector is organized into three main levels (central, intermediate and peripheral), each having specific competencies, administrative, health and dialogue structures [25]. The central level is headed by the Ministry of Public Health and focuses mainly on development of policies, strategies, and coordination. The intermediate level headed by the 10 regional delegations gives technical support to the 189 health districts nationwide. The health districts which are at the third level are the main implementation level for primary health care in Cameroon. Preventive services, including immunization services, are incorporated into all levels of the health system [26].

### 2.2. Sampling

Multistage sampling was used after allocating the number of districts and health facilities to be selected. The number of districts was allocated in proportion to the total number of districts per region, in the ten regions. Then, the number of urban and rural districts was assigned within each region based on the region-specific breakdown and health facilities allocated across regions in proportion to the national distribution.

The districts were then randomly selected within specified regions, urban or rural strata, in the first stage and health facilities were randomly selected within the identified rural/urban districts in the second stage. This was carried out while ensuring that the same number of facilities was selected within each district in a region. In each facility, five caregivers’ children brought for immunization were randomly selected for interviews. The following were used as inclusion criteria: caregiver aged above 21 years, caregiver (parents, guardians or family members) who regularly accompanied children to vaccination sessions. Caregivers were excluded if they declined consent.

### 2.3. Study Procedures

#### 2.3.1. Trainings

Trainings were conducted in all regions by the baseline assessment management team members to provide regional supervisors and assessors with the necessary knowledge and skills to undertake the baseline assessment. Assessors were carefully recruited with guidance from the regional delegations and local academic institutions, ensuring that they had prior experience in data collection and management. Training lasted for a minimum of two days and consisted of theoretical presentations and practical sessions on data collection, entry, and transmission processes.

#### 2.3.2. Data Collection

After developing a detailed data collection plan, assessors contacted health facilities via phone to remind them of planned visits in order to prevent unproductive visits. Upon arrival in each health facility, a fixed-post vaccination session was observed, and five caregivers (at least 21 years old) were randomly selected for interview.

Data were collected using a structured questionnaire, capturing basic sociodemographic information, caregiver experience and satisfaction with immunization services, and suggestions for improvement of services. The study questionnaire was developed in English and French, and pre-tested in four facilities in the Centre region prior to the study initiation.

#### 2.3.3. Data Management and Analysis

Each assessor entered data from the filled questionnaires into a database and transmitted the files to a secure server within three days of data collection. Data were exported to and cleaned in Microsoft Excel 2016 and analyzed with Stata 13 software (StataCorp. 2013. Stata Statistical Software: Release 13. College Station, TX, USA: StataCorp LP). Frequencies and proportions were used to summarize variables, and no post-stratification weights were applied since districts and facilities were sampled proportional to national distributions. Caregiver satisfaction with immunization services was assessed using a 4-point Likert scale (Very satisfied, Satisfied, Mixed feelings and Unsatisfied).

#### 2.3.4. Ethical Considerations

Prior to commencement of study, ethical approval was obtained from the National Ethics Committee, and administrative approvals were obtained from the Ministry of Public Health and the ten regional delegations of public health. The study was explained to participants in the most basic language possible explaining details of the study and informed consent (written or verbal) was obtained from all caregivers before data collection. Participants had the right to withdraw from the study at any time, and confidentiality was maintained by using codes instead of names on the data collection forms.

## 3. Results

Overall, 1230 caregivers were interviewed in 265 health facilities across 68 health districts. Slightly over half (53%) of these facilities were located in rural areas (Table 1). The median age of participants was 27 years and the median number of children per caregiver was two.

### 3.1. Vaccination Waiting Time

The median waiting time for vaccination was 1.8 h (1 h 48 min), with great regional variations. The median waiting time was greater than or equal to three hours in five regions: ES (3 h), EN (3.6 h), LT (3.1 h), NO (4.1 h) and OU (3 h). On the other hand, the median waiting time for caregivers was less than one hour in the SU (0.3 h) and NW (0.7 h) regions.

### 3.2. Utilization of Immunization Services

About a quarter (24%) of surveyed caregivers reported presenting to a health facility for immunization services and being turned away without achieving the purpose for which they came at least once. The percentage of respondents with such experiences was notably high in the OU (39%), NO (34%) and AD (32%) regions as summarized in Table 2.

As concerns outreach vaccination activities, a small percentage (4%) of caregivers reported to have shown up for an outreach activity that was canceled, and up to 48% of respondents had never heard about planned vaccination outreaches by health facilities in their respective communities.

### 3.3. Caregiver Satisfaction with Quality of Immunization Services

The majority (87%) of the respondents reported to be satisfied with the quality of the immunization services they received (Table 2), with 92% of caregivers stating that health care workers were friendly and nice to them during vaccination sessions.

### 3.4. Caregiver Suggestions for Improving Immunization Service Delivery

Some caregivers gave a few suggestions for improvement of the quality of immunization services. The most common suggestions voiced included the need to do the following:

- Address human resource constraints at the health facility level. Caregivers voiced the need to increase the number of care health workers to decrease the waiting time.
- Improve stock availability and adequacy at the facility level. Participants recommended facilities ensure that vaccines are available at the service delivery point on vaccination days.
- Improve the style of delivering health talks. For instance, caregivers indicated the need for facility staff to deliver health talks in simple and non-technical terms.
- Improve health facilities infrastructure to accommodate more caregivers.
- Increase the number of immunization outreach sessions to limit long-distance travel to the health facility.

## 4. Discussion

In the spirit of “leaving no one behind”, efforts need to be made to investigate every single factor that may impede a child from receiving his/her vaccinations, particularly in settings where coverage has been stagnant or slipping backward. Driven by this vision, we examined the immunization experiences of children’s caregivers in Cameroon, to identify potential barriers to timely and complete child vaccination. Based on our findings, the majority of caregivers expressed satisfaction with the immunization services provided. Nonetheless, there were some remarkable concerns raised including long waiting periods, inconsistencies in immunization service delivery, and insufficient communication regarding outreach activities.

Most (87%) of the respondents in our study reported to be satisfied with the immunization service quality. Our findings are similar to reports from Ethiopia (84.7%), India (91.9–95.9%) and Zambia (82.7%) [12,27,28]. However, the satisfaction rate in our study was higher than what was reported in other studies in Iraq (50.2%), Egypt (63%) and Ethiopia (68.2%) [29,30,31]. This variance could be due to real differences in immunization service quality, sociocultural differences and different techniques of assessing satisfaction, among other reasons. Understanding the level and reasons for client satisfaction with immunization services is key for the development of quality improvement strategies for immunization service delivery in Cameroon.

While many caregivers appeared to be satisfied with immunization services, some expressed resounding concerns including long waiting times. The median waiting time for vaccination was 1 h 48 min, with regional median waiting times ranging from 18 min in the South region to 4 h 6 min in the North region. This marked variation could be explained by the relative differences in health workforce and health facility densities and distributions over the national territory. For example, according to the Ministry of Public Health’s 2016 Health Map and 2018 Health Unit Profile, the South region had the highest number of health facilities per 10,000 population (3.71 in 2016 and 3.84 in 2018) as opposed to the three Northern regions that had the lowest ranging from 0.92 to 1.48 [32]. Long immunization waiting time is also most likely linked to health care workers’ attitudes and practices with regard to opening multi-dose vials (waiting for a certain number of children to be present before a vial is opened). Our findings are comparable to those reported by GebreEyesus et al. in Ethiopia [29] and Udonwa et al. in Nigeria [33], where 79% and 62.4% of health facilities, respectively, had a vaccination waiting time of greater than 30 min. Waiting time has been shown in multiple studies to be a major driver of caregiver satisfaction with childhood immunization services [12,33,34]. As such, exploring ways to shorten service waiting times may improve the immunization experience and enhance the utilization of immunization services in our setting.

In our study, about one in four caregivers (24%) reported presenting to a facility for immunization services and being turned away without achieving the purpose for which they came for at least once. This is higher than 14.7% reported in a similar national assessment in Zambia [12]. This practice, which may be linked to health workforce deficits, vaccine stockouts and the fact that many health facilities do not offer immunization daily, could contribute to missed opportunities for vaccination. This highlights the need to strengthen the vaccine supply chain and immunization workforce, expand immunization services in health facilities by offering them daily or at least more frequently, and improve communication between health facilities and caregivers to ensure that they are aware of the days and times when immunization services are available.

Our study also identified suboptimal community engagement in the planning and execution of outreaches. Indeed, about half (48%) of surveyed caregivers had never heard about planned vaccination activities in their communities, reaffirming the above claim. This finding highlights the need to strengthen community engagement in vaccination service delivery in Cameroon, as this is key for building trust in the health care system and narrowing inequities in immunization coverage.

Although our study has unveiled important barriers to optimal immunization service delivery from a caregiver perspective in Cameroon, certain limitations must be considered while interpreting the findings of our study. Caregiver interviews were conducted at the facility in the presence of health care workers; thus, it is possible that the data collected would be biased towards a favorable impression of the facility. Secondly, satisfaction was assessed only at a general level and may mask variations in satisfaction that exist when satisfaction is assessed in smaller domains (e.g., facility cleanliness, waiting time, health worker attitude, information dissemination and confidentiality). Additionally, only a small sample of caregivers were sampled in the health facilities and this may not be representative of the views of most caregivers in some settings. However, to the best of our knowledge, this is the first nationwide study assessing caregivers’ perspectives of immunization service delivery in Cameroon, while highlighting noteworthy regional differences. As such, addressing challenges faced by caregivers may go a long way to improve their experience with immunization services, which in turn may improve their acceptance of this cost-effective primary health care intervention and by extension, national immunization coverage in Cameroon.

## 5. Conclusions

While most caregivers appeared to be satisfied with immunization service delivery in Cameroon, some expressed noteworthy concerns including long waiting times, unproductive immunization visits and inadequate information about outreach activities. Investing in exploring and addressing these multi-faceted concerns, while conducting further studies to explore the root causes will go a long way to enhance the immunization experience of children’s caregivers in Cameroon, build trust in immunization services and thus improve vaccination uptake.

## Figures and Tables

**Table 1:** General characteristics of surveyed health facilities.

Regions ^a^	AD	CE	ES	EN	LT	NO	NW	SU	SW	OU	NAT
Distribution of health facilities (N)											
Rural (N)	8	22	14	10	12	8	26	19	8	13	140
Urban (N)	3	34	7	6	22	8	9	13	6	17	125
Total (N)	11	56	21	16	34	16	35	32	14	30	265

^a^ NAT National, AD Adamawa, CE Center, ES East, EN Extreme North, LT Littoral, NO North, NW North-West, SU South, SW South-West, OU West.

**Table 2:** Immunization experience of caregivers.

Region ^a^	AD	CE	ES	EN	LT	NO	NW	SU	SW	OU	NAT
	%	%	%	%	%	%	%	%	%	%	%
% of caregivers who showed up for services at a facility and were turned away											
Yes	32	24	31	24	21	34	14	27	11	39	24
No	68	77	69	76	79	66	86	73	89	61	77
% of caregivers who showed up for an outreach session that was canceled											
Yes	8	2	3	8	1	6	6	5	8	2	4
No	92	98	97	92	99	94	94	95	92	98	96
Frequency at which caregivers heard about outreach services being offered by their health facilities (%)											
Never	73	44	66	16	67	8	33	82	58	57	48
Monthly	27	38	24	51	5	49	51	10	20	7	30
Others (e.g., quarterly)	0	1	0	2	5	3	4	0	5	0	2
Don’t Know	0	0	7	0	14	12	11	8	16	11	8
Caregivers’ satisfaction with the quality immunization services at the facility (%)											
Very satisfied	16	18	22	37	33	20	36	33	49	40	31
Satisfied	81	60	68	50	57	57	55	57	44	49	56
Mixed feelings	3	7	3	9	4	12	2	8	6	7	6
Unsatisfied	0	12	7	3	5	5	6	3	1	1	6
Not reported	0	2	0	2	0	6	0	0	0	3	1

^a^ NAT National, AD Adamawa, CE Center, ES East, EN Extreme North, LT Littoral, NO North, NW North-West, SU South, SW South-West, OU West.

## Data Availability

Data used for this research are available from the corresponding author upon reasonable request.
